# Postoperative Hyperuricemia—A Risk Factor in Elective Cardiosurgical Patients

**DOI:** 10.3390/metabo13050590

**Published:** 2023-04-25

**Authors:** Dominik Raos, Ingrid Prkačin, Điđi Delalić, Tomislav Bulum, Martina Lovrić Benčić, Juraj Jug

**Affiliations:** 1 Institute of Emergency Medicine of Zagreb County, 10000 Zagreb, Croatia; 2 School of Medicine, University of Zagreb, 10000 Zagreb, Croatiajuraj2304@gmail.com (J.J.); 3 Department of Internal Medicine, Merkur University Hospital, 10000 Zagreb, Croatia; 4 Department of Diabetes, Vuk Vrhovac University Clinic for Diabetes, Endocrinology, and Metabolic Diseases, Merkur University Hospital, 10000 Zagreb, Croatia; 5 Department for Ischemic Heart Disease, University Clinic of Cardiovascular Diseases, Clinical Hospital Center Zagreb, 10000 Zagreb, Croatia; 6 Health Center Zagreb-West, 10000 Zagreb, Croatia

**Keywords:** hyperuricemia, mechanical ventilation, cardiac surgery

## Abstract

Hyperuricemia is a well-known cardiovascular risk factor. The aim of our study was to investigate the connection between postoperative hyperuricemia and poor outcomes after elective cardiac surgery compared to patients without postoperative hyperuricemia. In this retrospective study, a total of 227 patients after elective cardiac surgery were divided into two groups: 42 patients with postoperative hyperuricemia (mean age 65.14 ± 8.9 years) and a second group of 185 patients without it (mean age 62.67 ± 7.45 years). The time spent on mechanical ventilation (hours) and in the intensive care unit (days) were taken as the primary outcome measures while the secondary measure comprised postoperative complications. The preoperative patient characteristics were similar. Most of the patients were men. The EuroSCORE value of assessing the risk was not different between the groups nor the comorbidities. Among the most common comorbidities was hypertension, seen in 66% of all patients (69% in patients with postoperative hyperuricemia and 63.7% in those without it). A group of patients with postoperative hyperuricemia had a prolonged time of treatment in the intensive care unit (*p* = 0.03), as well as a prolonged duration of mechanical ventilation (*p* < 0.01) and a significantly higher incidence of the following postoperative complications: circulatory instability and/or low cardiac output syndrome (LCOS) (χ2 = 4486, *p* < 0.01), renal failure and/or continuous venovenous hemodiafiltration (CVVHDF’s) (χ2 = 10,241, *p* < 0.001), and mortality (χ2 = 5.22, *p* < 0.01). Compared to patients without postoperative hyperuricemia, elective cardiac patients with postoperative hyperuricemia have prolonged postoperative treatment in intensive care units, extended durations of mechanically assisted ventilation, and a higher incidence of postoperative circulatory instability, renal failure, and death.

## 1. Introduction

Uric acid (UA) as the main product of purine metabolism is formed from xanthine and hypoxanthine by the action of the enzyme xanthine oxidase [1,2]. The majority of daily purine turnover (about 60–70%) consists of endogenously synthesized purines and dietary ingested purines (about 30–40% of daily purine turnover). Purine bases in the liver are broken down into UA [2]. The main sources of exogenously introduced purine bases are poultry meat, fish and seafood, offal, meat products, yeast extracts, beer, alcohol, and from vegetables, peas, beans, and spinach [3]. Human cells do not have the ability to break down UA any further, and it is excreted from the body mostly through the kidneys (70%) and to a lesser extent through the digestive tract. In the state of renal insufficiency, the digestive system is the major route of UA excretion [4].

Hyperuricemia is an increase of UA concentration in serum, to a level above the physiological limit of 360 µmol/L (6 mg/dL) for women and more than 400 µmol/L (6.8 mg/dL) for men [5,6]. Serum UA level is susceptible to a variety of influences, including those with a genetic background (where potential racial/ethnic characteristics may be included), as well as cultural characteristics related to, for example, alcohol consumption and dietary characteristics [7].

The prevalence of hyperuricemia in the population is high (from about 5–8% to 25% of adult men in some areas) [8,9]. The prevalence of hyperuricemia in the general population in Croatia is 13.9% [10]. However, the annual incidence of gout, as a typical clinical manifestation of hyperuricemia, is 0.1% in a group of healthy individuals with a serum UA concentration below 416 μmol/L, 0.5% with a serum UA concentration between 416 and 529 μmol/L, and 4.9% in initially healthy individuals with a serum UA concentration higher than 535 μmol/L [11].

Hyperuricemia occurs due to increased urate synthesis (due to inherited defects in the regulation of purine nucleotide synthesis, ATP metabolism or due to increased cell breakdown) or their decreased renal excretion. Decreased renal excretion is responsible for 85–90% of primary or secondary hyperuricemia, and volume depletion is (due to diuretic therapy) one of the more common causes of hyperuricemia [6]. 

Almost 40 years ago, the role of xanthine oxidase and conversion to UA and free radical production in postischemic tissue injury was described and UA was assumed to be a marker of tissue damage in the acute setting [12]. A connection between hyperuricemia and higher cardiovascular risk has been confirmed in several studies [13,14,15,16]. Some emphasize the detrimental effect of UA on endothelial function, oxidative metabolism, and platelet aggregation, which can lead to endothelial dysfunction and vascular remodeling through oxidative–reductive stress [17,18]. UA could be a very sensitive marker of vascular wall inflammation, remodeling within the arterial wall, and capillary interstitium [19]. In addition, a connection was found between serum UA concentration and the incidence of peripheral vascular disease, metabolic syndrome, stroke, and renal disease [20,21]. Hyperuricemia has been confirmed as a risk marker for long-term outcomes after acute heart failure. Namely, in patients hospitalized for heart failure, increased serum UA concentration is associated with death or rehospitalization [22,23].

Coronary heart disease is a consequence of progressive coronary artery stenosis due to atherosclerosis [24,25,26]. Myocardial revascularization or coronary artery bypass grafting (CABG) is the most common cardiac surgery performed in the world today. After elective cardiac surgery, all patients are kept in an intensive care unit, sedated, and mechanically ventilated. In today’s conditions, machine ventilation usually lasts from six to twelve hours, while a prolonged time of machine-assisted ventilation increases the possibility of developing ventilator-associated pneumonia (VAP) [27]. EuroSCORE is a rating system for risk assessment in cardiac surgery for cardiac bypass placement and heart valve replacement [28]. 

### Aim of the Study

Hyperuricemia is associated with a less favorable outcome in patients following elective cardiac surgery. The aim of our study was to investigate the duration of mechanical ventilation after the procedure and the duration of stay in an intensive care unit dictated by the patient’s condition in elective cardiac surgery patients with postoperative hyperuricemia. In addition, secondary outcome measures include the incidence of other postoperative complications: hemorrhage, surgical revisions, reintubation, acute renal failure, delirium, low cardiac output syndrome (LCOS), renal failure, and mortality.

## 2. Methods

We conducted a retrospective prognostic study on elective cardiac surgery patients. Over a period of four full years after elective cardiac surgery, the parameters of patients with documented postoperative hyperuricemia were compared with the parameters of patients without postoperative hyperuricemia. The study included 227 patients. Inclusion criteria were the availability of demographic (age and gender), anthropometric (body weight and height) and anamnestic data (arterial hypertension, diabetes, and hypercholesterolemia), laboratory findings of pre- and postoperative serum UA concentration, and documented cardiac surgery. The analyses were approved by the institutional ethics board of the University Hospital Merkur and the study was conducted in accordance with the Declaration of Helsinki and the good clinical practice guidelines.

### 2.1. Groups Depending on Urate Status

The patients were divided into two groups. The first group were those with postoperative hyperuricemia (more than 360 μmol/L or 6 mg/dL for women and more than 400 μmol/L or 6.8 mg/dL for men) (*n* = 42), and the second group were patients without postoperative hyperuricemia (*n* = 185).

### 2.2. Exclusion Criteria

Patients with incomplete documentation as well as patients under 45 years of age and over 75 (the available data suggest that ages outside these limits can have a significant impact on the outcome of the procedure) [29]. 

### 2.3. Other Procedures

Anthropometric, clinical, and laboratory parameters were recorded for all patients included in the study. From the personal history, the following data were recorded: age, gender, weight, and height, based on which the body mass index (BMI) was calculated, as well as previous diseases (arterial hypertension, diabetes, and hypercholesterolemia). The value of the risk assessment EuroSCORE (European System for Cardiac Operative Risk Evaluation) was also recorded. Postoperatively, values from the patient list of the intensive care unit were recorded. Preoperative laboratory tests were conducted as part of the preoperative evaluation of patient candidacy for the planned surgical procedure according to the hospital protocol (morning fasting peripheral venous blood samples taken and analyzed in the hospital laboratory on the same day). Postoperative laboratory tests were conducted immediately following the acquirement of peripheral venous and arterial blood samples in the hospital laboratory. As primary outcome measures, we took the time spent on mechanical ventilation (hours) and the time spent in the intensive care unit until the patient was evaluated as ready for transfer to the general surgery ward by the attending anesthesiologist (days). Logistically, it is important to clarify that the center at which the study was conducted has a separate surgical intensive care unit (SICU) tasked with managing surgical patients exclusively and does not admit trauma patients or patients from the emergency department; therefore, patient turnover is mostly independent of logistical limitations such as the deficiency of available beds in either the SICU or the general surgery ward, which is exclusively tasked with postoperative monitoring of stable patients not requiring SICU-level care. Secondary outcome measures included postoperative complications: bleeding, surgical revision, hypertension, renal failure, reintubation, low cardiac output syndrome (LCOS), delirium, and death.

Revision includes surgical re-exploration of the operating field (re-sternotomy) most often due to surgical bleeding greater than 500 mL/h in the first hour after surgery, greater than 400 mL/h in the second hour after surgery, greater than 300 mL/h in the third hour after surgery, or greater than 200 mL/h in any period thereafter. Revision is undertaken to prevent cardiac tamponade.

Renal failure was defined by using the criteria for acute kidney injury (AKI) from the KDIGO Clinical Practice Guideline for Acute Kidney Injury. 

Postoperative delirium was defined by using the definition from the Diagnostic and Statistical Manual of Mental Disorders, Fifth Edition (DSM-5), according to the recommendations by the European Society of Anaesthesiology (ESA). 

Hemorrhage was defined as a reduction in serum hemoglobin concentration of such a magnitude that it corresponded to the “transfusion triggers” described by the ESA guidelines for the management of severe perioperative bleeding [30].

LCOS was defined as a Cardiac Index (CI) of less than 2.0 L/min/m^2^. In this study, all CI values were obtained by the thermodilution method, since all patients had a pulmonary arterial catheter. All participants signed an informed consent form to participate in the research.

### 2.4. Statistical Analysis

Category variables are shown as proportions, and metrics are shown as arithmetic mean and standard deviation (X ± SD). The T test was used to determine a statistically significant difference between the numerical parameters. The correlation between categorical variables was determined by the χ2 test with Yates correction. The results are expressed as the value of *p*. The level of statistical significance was determined as *p* < 0.05. As the main outcome measure, we took the days spent in the intensive care unit; the preliminary measurements gave us a value of 5 days in the group without postoperative hyperuricemia and 7 days in the group with postoperative hyperuricemia, with a standard deviation of about 3 days. With a significance level of 0.05 and a study strength of 0.8, the program provided the size of each group of 36 subjects for the organization of the study from two groups of independent data.

## 3. Results

The preoperative characteristics of the patients did not differ significantly. All of the patients included in the study were normouricemic before surgery (serum UA levels within reference intervals) and also had a normal renal function determined with a creatinine-based estimated glomerular filtration rate (eGFR) of at least 60 mL/min/m^2^. Men were more numerous (149 men vs. 36 women). The measured preoperative values of serum UA in patients with postoperative normouricemia and postoperative hyperuricemia are presented in Table 1. The median age of patients with postoperative hyperuricemia was 65.14 ± 8.9, and of the group without elevated postoperative serum UA, the median age was 62.67 ± 7.45 years. The EuroSCORE value of the risk assessment did not differ significantly between the groups. There was no significant difference in morbidity between the groups. The most common was arterial hypertension, which affected 69% of patients in the group with postoperative hyperuricemia and 63.7% of patients in the group without postoperative hyperuricemia. All the above data are presented in Table 2.

Postoperative values of serum UA concentration were significantly higher in patients with postoperative hyperuricemia compared to patients with postoperative normouricemia (Table 3). In addition, there was a significantly higher percentage change in serum UA concentrations in patients with postoperative hyperuricemia compared to patients with postoperative normouricemia (Table 4).

After cardiac surgery, patients in the intensive care unit were connected to mechanical ventilation and hemodynamically monitored by comparing the metric parameters of patients with and without postoperative hyperuricemia (Table 5).

Our research shows that the treatment time in the intensive care unit differed significantly between the groups. The group of patients with postoperative hyperuricemia had a statistically significantly prolonged treatment time in the intensive care unit compared to the group of patients without postoperative hyperuricemia (*p* = 0.0263). Time spent on machine-assisted ventilation also differs significantly between the two groups. The group of patients with postoperative hyperuricemia has a significantly longer duration of mechanical ventilation compared to patients with postoperative normouricemia (*p* = 0.0036) (Table 5). A significantly higher incidence of LCOS (χ2 = 4.486; *p* = 0.034), renal failure and/or continuous venovenous hemodiafiltration (CVVHDF) (χ2 = 10.241; *p* = 0.001), and mortality (χ2 = 5.22; *p* = 0.021) was observed in the patient group with postoperative hyperuricemia.

LCOS was observed in 47.6%, renal failure and/or CVVHDF were observed in 33.3%, and mortality was observed in 7.1% of patients with postoperative hyperuricemia. Of the 20 patients with postoperative hyperuricemia and LCOS, 8 of them also required renal intervention in the form of hemodialysis. No significant difference in other postoperative complications was found between the groups of patients (Table 6). 

## 4. Discussion

The results of our study showed that compared to patients without postoperative hyperuricemia, in the group of elective cardiac surgery patients with postoperative hyperuricemia, the treatment time in the intensive care unit was prolonged as well as the time spent on machine-assisted ventilation. Furthermore, patients with postoperative hyperuricemia are significantly more likely to be prone to circulatory instability and renal failure, and ultimately death. In the available literature, we found the oldest and most published study like ours—the Framingham study, which included 6763 patients and for the first time indicated the association of hyperuricemia with an increased incidence of cardiovascular diseases in the general population. However, this association was not independent of hypertension or other similar risk factors for the occurrence of cardiovascular disease [31]. Another major epidemiological study (National Health and Nutrition Examination Survey I, NHANES I) (5926 subjects, mean follow-up 16.4 years), published in 2000, showed that hyperuricemia was an independent risk factor for cardiovascular mortality in the general population [32]. In contrast to our study, where patients ranged in age from 45 to 75, the NHANES I study reported the highest risk in the male and female population between the ages of 45 and 54. The results of these two studies are in line with our results.

Recently, serum UA has risen to prominence as a prospective predictor of various adverse events following cardiac surgery and has been studied in the contexts of several different cardiac surgery procedures, the most important of which are listed and discussed below. Lazzeroni et al. investigated the role of serum UA concentrations in predicting adverse outcomes following surgical myocardial revascularization. They found that patients with elevated UA serum concentrations were at significantly higher risk for cardiovascular mortality [hazard ratio (HR) = 2.0, 95% confidence interval (CI) 1.2–3.2, *p* = 0.004], major adverse cardiac and cerebrovascular events (HR = 1.5, 95% CI 1.0–2.0, *p* = 0.019), and overall mortality (HR = 2.1, 95% CI 1.5–3.0, *p* < 0.001), after adjustment for arterial hypertension, diabetes, glomerular filtration rate, age, gender, atrial fibrillation, and medical therapy [33]. Shi et al. found similar results while studying patients who underwent a coronary artery bypass graft (CABG). During a three-year follow-up period, patients with elevated serum UA concentrations were at significantly higher risk of major adverse cardiovascular events (MACE) (HR = 1.70, 95% CI 1.12–2.57; *p* = 0.01) and a composite endpoint of mortality or myocardial infarction (HR = 2.42, 95% CI 1.32–4.43, *p* = 0.004) [34].

Several groups of authors have studied the importance of serum UA concentrations in predicting adverse outcomes following Stanford Type A aortic dissection repair (STAADR). Ma et al. found that serum UA concentrations on postoperative day 1 were independent predictors of 30-day postoperative mortality in patients undergoing this type of procedure [odds ratio (OR) 2.562, 95% CI 1.635–4.014, *p* < 0.001) with an area under the curve (AUC) of 0.799 [35]. Yang et al. found a weak, but significant correlation between serum UA concentrations on admission and in-hospital mortality following STAADR (OR = 1.04, 95% CI 1.02–1.06) [36]. UA may also play a role in heart transplant rejection, as Asleh et al. found that elevated serum UA concentrations are independently and significantly correlated with an increased risk of cardiac allograft vasculopathy (HR 2.2, 95% CI 1.1–4.6, *p* = 0.037) [37].

One of the areas in which the role of UA as a predictive factor has been well researched is the development of acute kidney injury (AKI) following cardiac surgery. Kaufeld et al. identified elevated preoperative serum UA concentrations as an independent risk factor for the development of postoperative AKI in cardiac surgery patients (OR 5.497, 95% CI 1.772–17.054, *p* = 0.003) [38]. Su et al. also found that serum UA concentrations were an independent risk factor for the development of AKI post surgery (OR = 1.237, 95% CI 1.095–1.885 *p* = 0.009) [39]. UA has also been proven useful as part of predictive models that include multiple variables. Hu et al. found that a model that included age, male gender, left ventricular ejection fraction, hypertension, hemoglobin, serum UA concentrations, hypomagnesemia, use of oral renin–angiotensin system inhibitors, and the use of non-steroidal anti-inflammatory drugs within 1 week before surgery predicted the development of postoperative AKI with an AUC of 0.740 [40]. The same group of authors also constructed a similar model for predicting postoperative AKI in elderly patients. This model included preoperative serum creatinine, history of hypertension, preoperative serum UA concentration, New York Heart Association classification ≥ 3, cardiopulmonary bypass time > 120 min, intraoperative red blood cell transfusion and prolonged postoperative mechanical ventilation; it has been shown to predict postoperative AKI in patients aged ≥ 60 years with an AUC of 0.801 [41]. Pan et al. also developed a model for predicting the development of AKI following cardiac valve replacement surgery. Their model includes age, hemoglobin, fibrinogen, serum UA concentration, cystatin C, serum bicarbonate, and cardiopulmonary bypass time and predicts the development of postoperative AKI with an AUC of 0.760 [42]. Lastly, a study by Fan et al. utilizing machine learning and biomarkers in order to develop a predictive model for AKI following cardiac surgery found that serum UA concentration predicts postoperative AKI with an AUC of 0.749 [43].

Our study is in line with the findings listed above—elevated postoperative serum UA levels are a risk factor in cardiovascular surgery patients. It is important to emphasize that in the selection of patients for this study, we introduced age as the inclusion criterion precisely to avoid its impact on the value of the EuroSCORE and the possible impact on the overall cardiovascular risk. The group of patients with documented postoperative hyperuricemia had a significantly prolonged treatment time in the intensive care unit, as well as the length of stay on machine-assisted ventilation. In addition, two very serious postoperative complications were more commonly reported in the same patients: circulatory instability and renal failure. Death, as the final treatment outcome, was also more frequently reported in patients with postoperative hyperuricemia. Since there was no difference between our two groups of patients in other cardiovascular risk factors (age, gender, elevated BMI, hypertension, diabetes, and hyperlipidemia), the results obtained in our study show a direct association of elevated postoperative serum UA with a poor treatment outcome and more frequent postoperative complications in cardiovascular surgery patients.

Besides death and renal failure, discussed above, our study also examined the correlation between elevated postoperative serum UA concentrations and postoperative delirium. Postoperative delirium was chosen as one of the secondary study outcomes due to it being a relatively common postoperative complication in major cardiovascular surgery: studies report an incidence between 5 and 39%, depending on the patient age and procedure type [44]. There is evidence that delirium is independently associated with both an increased risk of death (HR 3.2, 95% CI 1.4–7.7, *p* = 0.008) and a longer hospital stay (HR, 2.0, 95%CI 1.4–3.0, *p* < 0.001) [45]. There is discordant evidence regarding the association of serum UA concentrations with postoperative delirium, with some authors finding low serum UA concentrations to be associated with delirium, while others found the same for high serum UA concentrations. Xu et al. found a protective effect of high preoperative UA serum concentrations (*p* = 0.040) regarding the development of postoperative delirium [46]. Liu et al. found that a lower serum UA concentration to serum creatinine concentration (SUA/SCr) ratio significantly increases the risk of postoperative delirium (*p* < 0.001) [47]. Wang et al. found elevated serum UA concentrations as a risk of postoperative delirium (*p* = 0.031) in patients undergoing orthopedic surgery. [48]. Sharma et al. also confirmed that patients with delirium had significantly higher serum UA concentrations (*p* = 0.014) [49]. The results of our study show no statistically significant differences in postoperative serum UA concentrations between patients with and without postoperative delirium.

Our study also found a significantly higher incidence of post-operative LCOS in patients with postoperative hyperuricemia. This is a very important finding, as LCOS is a significant diagnostic and therapeutic challenge in both pediatric and adult cardiac surgery. Duncan et al. found that LCOS presents a significant source of costs and complications; patients with postoperative LCOS have a significantly higher risk of in-hospital mortality (OR 12, 95% CI 10.6–13.5), incur significantly higher medical care costs (average hospitalization cost USD 64,041 for patients with LCOS versus USD 48,086 for patients without LCOS, *p* < 0.001), and have significantly higher hospital readmission rates (16.6% vs. 13.9%, *p* < 0.001) [50]. Maganti et al.’s research found similar results: patients with postoperative LCOS had a significantly higher mortality than patients without (30% vs. 1.3%, *p* < 0.001). Independent predictors of increased mortality in patients with LCOS were renal failure (OR 4.3), patient age (OR 1.03) and the need for a reoperative surgical intervention (OR 1.8) [51]. Further studies should be carried out on the utility of both pre and postoperative hyperuricemia in predicting LCOS, as there is evidence that adjusting the type of cardiac surgery procedure and using inotropes such as levosimendan postoperatively significantly reduce the risk of postoperative LCOS [52]. Recognizing the patients at risk of this major cardiac surgery complication before the procedure itself or immediately after using a single noninvasive blood biomarker (UA) would have the potential to significantly reduce in-hospital mortality, hospitalization costs, and complication rates. 

### Strengths and Limitations

The strength of this study is that the data were collected on a variety of anthropometric, laboratory, and clinical parameters. This has enabled the identification of potential risk factors for poorer patient outcomes besides urate, while also allowing the authors to determine the dependence of the predictive value of serum UA concentrations on other factors such as age, sex, comorbidities etc. In addition, following statistical data analysis, postoperative hyperuricemia was successfully singled out and identified as an independent predictive factor for worse primary and secondary outcomes. In this study, we used serum UA concentration. The serum or plasma matrix should generate similar results in clinical and biological studies. However, it seems that higher metabolite concentrations in serum make it possible to provide more sensitive results in biomarker detection [53].

This study has several limitations. The first limitation is that this is a single center study in a tertiary care clinic, which may limit the diversity or representativeness of this study’s patient pool. The second limitation is that the number of patients was relatively small. The third limitation is the fact that the study is retrospective, which might affect the reliability of the data obtained retrospectively and bias the results.

## 5. Conclusions

The results presented in this study suggest that there is a potential role for the measurement of serum UA concentration at patient admission in risk assessment systems in patients with cardiovascular disease, or at least in some subgroups of patients, especially keeping in mind that this is a simple, reliable, and cheaply measurable indicator, although a small sample size and the fact that it is a single-centre study limit the generalizeability of this recommendation. 

## Figures and Tables

**Table 1:** Measured preoperative values of serum uric acid concentration.

Patient Group	Mean Serum Uric Acid Concentration (umol/L) +/− SD (Split by Gender)	Mean Serum Uric Acid Concentration (μmol/L) (without Gender Split)
Postoperative normouricemia (*n* = 185)	312 +/− 9 (Females, *n* = 36)	343.5
	376 +/− 12 (Males, *n* = 149)	
Postoperative hyperuricemia (*n* = 42)	351 +/− 8 (Females, *n* = 6)	372
	394 +/− 11 (Males, *n* = 36)	

SD—standard deviation; *n*—number of patients.

**Table 2:** Postoperative characteristics of elective cardiac surgery patients treated in the intensive care unit.

Demographic and Anamnestic Data	Postoperative Normouricemia (*n* = 185)	Postoperative Hyperuricemia (*n* = 42)	*p* *
Gender (M/F)	149/36	36/6	0.515
Age (X ± SD)	62.67 (7.45)	65.14 (8.09)	0.057
BMI ^†^ (Kg/m²) (X ± SD)	27.70 (3.50)	28.47 (3.67)	0.203
EuroSCORE ^‡^ (X ± SD)	3.87 (3.01)	4.64 (3.57)	0.152
Diabetes (number, %)	50 (27.02)	12 (28.57)	0.849
Hyperlipidemia (number, %)	124 (67.02)	25 (59.52)	0.372
Arterial hypertension (number, %)	118 (63.78)	29 (69.04)	0.594

* *T*-test and χ2-test. ^†^ BMI = Body mass index. ^‡^ EuroSCORE = European System for Cardiac Operative Risk Evaluation.

**Table 3:** Measured postoperative values of serum uric acid concentration.

Patient Group	Mean Serum Uric Acid Concentration (μmol/L) +/− SD (Split by Gender)	Mean Serum Uric Acid Concentration (μmol/L) (without Gender Split)
Postoperative normouricemia (*n* = 185)	321 +/− 8 (Females, *n* = 36)	351.5
	382 +/− 11 (Males, *n* = 149)	
Postoperative hyperuricemia (*n* = 42)	397 +/− 12 (Females, *n* = 6) *	414 *
	431 +/− 11 (Males, *n* = 36) *	

SD—standard deviation, *n*—number of patients, *—*p* < 0.05.

**Table 4:** Measured percentage change in serum uric acid concentrations postoperatively compared to preoperative values.

Patient Group	Percentage Change of Mean Serum Uric Acid Concentration (%) (Split by Gender)	Percentage Change of Mean Serum Uric Acid Concentration (%) (without Gender Split)
Postoperative normouricemia (*n* = 185)	+2.80 (Females, *n* = 36)	+2.28
	+1.57 (Males, *n* = 149)	
Postoperative hyperuricemia (*n* = 42)	+11.59 (Females, *n* = 6) *	+10.14 *
	+8.58 (Males, *n* = 36) *	

*n*—number of patients; *—*p* < 0.05.

**Table 5:** Comparison of postoperative metric parameters of the patients.

	Postoperative Normouricemia (*n* = 185)	Postoperative Hyperuricemia (*n* = 42)	p *
Treatment duration in ICU (days)	4.38 ± 1.95	5.45 ± 5.07	0.026
Duration of mechanical ventilation (hours)	17 ± 14.47	27 ± 35.97	0.004

* *T*-test.

**Table 6:** Comparison of postoperative categorical parameters of elective cardiac surgery patients treated in the intensive care unit.

Analyzed Parameter	Postoperative Normouricemia (*n* = 185)	Postoperative Hyperuricemia (*n* = 42)	χ2	p *
Hemorrhage	22/185 (11.8%)	7/42 (16.6%)	0.337	0.442
Revision	11/185 (5.9%)	3/42 (7.1%)	0.000	1.000
Delirium	17/185 (9.1%)	5/42 (11.9%)	0.062	0.804
Hypertension	19/185 (10.2%)	2/42 (4.7%)	0.668	0.414
LCOS ^†^	54/185 (29.7%)	20/42 (47.6%)	4.486	0.034
Reintubation	3/185 (1.6%)	2/42 (4.7%)	0.383	0.536
Renal failure and/or CVVHDF ^‡^	22/185 (11.8%)	14/42 (33.3%)	10.241	0.001
Death	1/185 (0.5%)	3/42 (7.1%)	5.22	0.021

* χ2-test with Yates correction. ^†^ LCOS = Low cardiac output syndrome. ^‡^ CVVHDF = Continuous veno-venous hemodialysis.

## Data Availability

The data presented in this study are available from the corresponding author upon request. The data are not publicly available due to privacy or ethical.
